# Patient preferences for participation in patient care and safety activities in hospitals

**DOI:** 10.1186/s12912-017-0266-7

**Published:** 2017-11-21

**Authors:** Mona Ringdal, Wendy Chaboyer, Kerstin Ulin, Tracey Bucknall, Lena Oxelmark

**Affiliations:** 1 Institute of Health and Care Sciences, The Sahlgrenska Academy, University of Gothenburg, Box 457, 40530 Gothenburg, SE Sweden; 2 Department of Anaesthesiology and Intensive Care, Kungälvs hospital, Kungälv, Sweden; 3 National Centre of Research Excellence in Nursing Interventions (NCREN) Menzies Health Institute, School of Nursing, Griffith University, Gold Coast, Australia; 4 Department of Medicine, Sahlgrenska University Hospital, Gothenburg, Sweden; 5 School of Nursing and Midwifery, Deakin University, Geelong, Victoria, Australia; 6 Deakin University’s Centre for a Quality and Patient Safety-Alfred Health Partnership, Melbourne, Victoria, Australia

**Keywords:** Nursing, Communication, Participation, Patient-centred care, Patient safety, Patient engagement, Patient involvement, Patient participation, Shared decision-making

## Abstract

**Background:**

Active patient participation is a patient safety priority for health care. Yet, patients and their preferences are less understood. The aim of the study was to explore hospitalised patients’ preferences on participation in their care and safety activities in Sweden.

**Methods:**

Exploratory qualitative study. Data were collected over a four-month period in 2013 and 2014. Semi-structured interviews were conducted with 20 patients who were admitted to one of four medical wards at a university hospital in Sweden. Data were analysed using thematic analysis.

**Results:**

Nine men and eleven women, whose median age was 72 years (range 22–89), were included in the study. Five themes emerged with the thematic analysis: endorsing participation; understanding enables participation; enacting patient safety by participation; impediments to participation; and the significance of participation. This study demonstrated that patients wanted to be active participants in their care and safety activities by having a voice and being a part of the decision-making process, sharing information and possessing knowledge about their conditions. These factors were all enablers for patient participation. However, a number of barriers hampered participation, such as power imbalances, lack of patient acuity and patient uncertainty. Patients’ participation in care and patient safety activities seemed to determine whether patients were feeling safe or ignored.

**Conclusion:**

This study contributes to the existing literature with fundamental evidence of patients’ willingness to participate in care and safety activities. Promoting patient participation begins by understanding the patients’ unique preferences and needs for care, establishing a good relationship and paying attention to each patient’s ability to participate despite their illness.

## Background

Patient safety has been a key focus of international governments and healthcare providers for more than a decade. The focus on patient safety as a risk management strategy is associated with increasing accountability and transparency in the healthcare decision-making process [1]. As part of this movement, leaders have called for greater patient participation in healthcare. Attributes of promoting patient participation include establishing a relationship between nurses and patients, decreasing nurse control, sharing information and knowledge between nurses and patients and finally actively engaging patients in both intellectual and physical activities [2]. Yet, the extent to which, and in what ways, patients want to and are able to contribute remains complicated and multifaceted.

The term participation is used to describe both physical and verbal forms of patient involvement in hospital care. Patient participation in care has been examined from both nurses’ and patients’ perspectives [3]. Nurses suggest a fundamental shift in how to provide care [4], and they also think that patient participation in care and team meetings is an unfamiliar situation for the patient [5]. Research has found that patients view nurses’ behaviour as both promoting and inhibiting to active participation [6].

A recent Swedish report found that patients in Sweden gave low scores on accessibility, information and patient participation, showing no improvement over the last 5 years [7]. A particular interest in patients’ participation in safety activities, such as medication administration and hand-washing, occurred on some wards but not all [8]. Furthermore, in Sweden, patients did not believe that care providers shared all key information with them [9]. Evidence suggests that patient participation improves many aspects from patient satisfaction [10, 11], patient trust in clinicians [12], patient adherence to treatments [13] to patient outcomes [14, 15]. Participation also decreases healthcare utilisation [16] and prevents adverse events [10] and subsequent medical litigation from occurring [17]. Intuitively, a healthcare system characterized by patient-centeredness also supports patient participation in safety initiatives.

Although there is an increasing body of research that recognizes patient involvement is pivotal in healthcare reforms, and the Swedish National Board of Health and Welfare [18] highlights the importance of patient participation in care, opportunities to promote authentic patient participation are in their infancy. Other challenges, specifically involving nursing care, relate to the patients’ willingness, expectations and potential roles in care are left ambiguous [19].

It is likely that without all the important information, patients may struggle to participate in their own care actively. However, Vaismoradi et al. [3] argues that patients can easily participate in safety initiatives regardless of other factors. Yet, understanding patient preferences for participation in care and safety activities is not well understood.

### Aim

The aim of the study was to explore hospitalised patients’ preferences on participating in care and patient safety activities in Sweden.

## Methods

### Design

An exploratory study was conducted using semi-structured interviews with hospitalised patients.

### Settings and participants

The study took place in four medical wards in two hospitals that were both part of a university hospital setting. The two hospitals were geographically diverse, but in the same city in Sweden, admitted similar kinds of medical patients, although the catchment areas varied in terms of socioeconomic patient profiles. One of the hospitals practice person-centred care the other had ordinary care. The wards were selected to increase the heterogeneity of information for a better in-depth understanding of patients’ preferences. Adult patients with chronic conditions admitted to a medical ward for at least 3 days, with one or more co-morbidities, were invited to participate in the study. The participants were purposely selected in regards to age, gender and medical condition to gain a wide variation in patient experience [20]. A designated nurse in each ward approached potential participants with verbal and written information about the study. If the patients were willing to participate, a consent form was signed, and a time was scheduled for the interview.

### Data collection

Data were collected over 4 months in 2013 and 2014 by two of the researchers (LO, MR) using semi-structured interviews. An interview guide was used to ensure consistency. Examples of interview questions were: Can you tell me what participating in your own care means to you? When you participate in your care, what are you hoping to achieve? What interest do you have in participating in safety activities when you are being cared for in the hospital? What could facilitate or prevent you from participating in your care? The interviews took place at a time and place mutually convenient for the patient and researcher; either at the patients’ bedside or in a quiet room on the ward prior to the patient’s hospital discharge. Demographic data were also collected during the interview. The interviews were digitally recorded and transcribed verbatim.

### Data analysis

Thematic analysis [21] was used to analyse the data. Analysis was iterative and included a line-by-line analysis of the transcripts. Transcripts were first coded and grouped into subthemes and then themes through a process of pattern matching and refinement. Subthemes and their inter-relationships were re-examined and revised in a recursive manner in order to identify themes. Analysis continued until no new themes emerged, and there was researcher agreement on the themes. Finally, the themes, with their subthemes, were considered in relation to enablers, barriers and significance. The software program NVivo 10.0 was used to organise the text and manage the data. To enhance reliability, two researchers (MR, LO) read the transcripts independently and then together organised and coded the text with NVivo 10.0. Several meetings with different members of the research group enabled consensus to be reached in the final analysis and coding of the text.

### Ethical considerations

The study was approved by the Ethical Review Board at the University of Gothenburg (2013–10-04 Dnr 693–13). Participants were given both written and verbal information about the study. They were also informed that participation was voluntary, and they could withdraw from participation at any time without any consequences.

### Results

The sample consisted of 20 participants (9 men and 11 women) from four different medical wards. The median age of participants was 72 years (range 22–89). The median length of hospital stay was 5 days (range 3–14). Co-morbid conditions included cardiac disorders, diabetes mellitus, chronic obstructive pulmonary disease and various types of cancer.

Five themes were identified: endorsing participation; understanding enables participation; enacting patient safety by participation; impediments to participation; and the significance of participation. Ten subthemes are shown in the text with translated quotes italicised. The first three themes with their subthemes emerged as enablers, the fourth theme plus its subtheme as a barrier and the final theme and subtheme as significance (see Table 1).

**Table 1:** Themes and subthemes for patient participation

Endorsing participation	Having a voice	To be a part of the discussions and decision- making process	ENABLERS
Understanding enables participation	Possession of knowledge	Being informed	
Enacting patient safety by participation	Sharing information	Participating in specific safety activities	
Impediments to participation	Power imbalance	Being passive	BARRIERS
Significance of participation	Feeling safe	Feeling ignored	SIGNIFICANCE

First three themes and subthemes are enablers to participation and fourth theme with subthemes are barriers to participation. Last theme with subthemes are the significance of participation

### Endorsing participation

Participants wanted to participate in their own care, as they felt it gave them the opportunity to communicate their needs and how they felt to the healthcare professionals (HCPs).

#### Having a voice

Participants suggested that communication with nurses was a way to promote participation, as they could talk about their needs and feelings. Participation kept participants informed earlier about planned treatments, which allowed them to prepare better for upcoming treatments or events. It also gave them the opportunities to discuss care plans with the nurses. It was important for participants to have a voice***,*** to speak up and ask questions to gain a better understanding of their overall medical situation.

*You simply have to speak for yourself, attract attention all the time.*

Some of the participants saw themselves as partners in their care. They described how the information they shared with HCPs about their condition could correct misunderstandings or inaccuracies.

*It’s teamwork and you understand each other. You are allowed to speak up, and they actually listen to you.*

#### To be part of the discussions and decision-making process

Most participants wanted to be part of the discussions and decision making about their care, treatments and therapies. They were not satisfied with a passive role. Participants described how they were the experts in terms of their condition and wanted to ensure HCPs listened to them when they described their symptoms and experiences. In addition, they wanted the ability to approve or reject planned treatments and therapies.

*They can never do anything actually, without my approval.*

### Understanding enables participation

This theme reflected participants’ wishes to understand the implications of their illnesses and how understanding linked with their ability to participate in their care.

#### Possession of knowledge

Having knowledge about their illnesses makes it easier to discuss them. Participants’ actual possession of knowledge about their sickness facilitated participation towards their care. This knowledge came from different information sources. They sought information from media, scientific publications and the Internet searches to increase their knowledge about their condition. Additionally, they received information from other patients and from medical staff during rounds. Participants who had knowledgeable relatives also relied heavily upon them.

*I had a little help from my daughter, she knew about medicine.*

#### Being informed

It was important for participants to be informed about their disease, safety issues, such as pressure ulcers, various laboratory tests, and future care plans. They wished for general information to better understand what was happening to them. Some were disappointed to have received inadequate information. Participants stressed that physicians should volunteer information without patients having to ask. Therefore, being well informed facilitated participating in care.

*I should be informed about what is going to happen to me when I am sick and test results, and then I could be part of the decision-making.*

### Enacting patient safety by participation

All participants trusted the HCPs to protect them from risks and maintain their safety, but they also described that taking responsibility for their own safety was important, for example, by understanding their treatments and symptoms.

#### Sharing information

Participants wanted to share information with the staff about their long-term illnesses and how they wanted to be treated. Participants thought they possessed the best understanding of their own body.

*I know my body best, I know my history.*

Participants thought they participated in care and safety activities by adding information about their symptoms and checking the information about what to do about it in the written care plan that the nurses created.

*They are writing it down, the nurse-patient conversation, and you have that paper with you when you go home.*

#### Participating in specific safety activities

In terms of patient safety, participants often referred to participation in specific safety activities rather than in broad terms. Examples included requesting bedside rails and ensuring that the healthcare practitioners washed their hands before conducting a physical examination.

*If they do not clean their hands, I will point it out.*

Another area of specific safety participation was checking that they, the patients, received the right medication. Participants recognised the importance of the nurse checking their identity before dispensing their medications. If this check failed to occur, some participants intervened and showed their identity bracelet. Participants thought the identity bracelet every patient wore was crucial for safety, and they described how nurses checked their identity before performing care activities. Other specific examples were when participants checked their bandages if they became unclean or were not inspected by HCPs. Participants were knowledgeable about the importance of preventing pressure ulcers and wanted to prevent them by, for example, moving in bed, observing and taking care of the skin and trying to get out of bed as soon as possible. Some also knew to notify HCP once they felt any pressure points on their body. A few participants even discussed new technology, such as special mattresses, that could help prevent pressure ulcers.

*My date of birth and name are on the medication cup when they give it to me, so I know that it is my medication, and I know exactly what medication I am on……another example is when they give me a blood transfusion, they ask for my security number.*

### Impediments to participation

It was evident that even if participants wanted to participate in care and safety activities, they faced a number of challenges or issues. Impediments were described in relation to HCPs’ unwillingness to listen and/or to a bullying manner.

#### Power imbalance

Lacking knowledge and understanding of their medical condition hindered participation as did the power imbalance between HCPs and patients. Participants felt that a power imbalance existed when the HCPs were talking over their heads. Some participants complained of being on the outside, feeling unwelcome and not listened to which impeded their participation and a feeling of inferiority.

*I asked for a 7.5-mg sleeping pill, which I know makes me sleep. But they still give me a 5-mg pill, and I had to call for another one. “You try”, they said, “it works!” But I know after 50 times at the hospital that it doesn’t work. So why couldn’t they give me a 7.5 mg in the first place? This has been going on for three nights in a row now.*

#### Being passive

Being passive in their care often reflected participants’ weakness and fatigue due to their acute illness, not being able to take care of themselves and instead relying on HCPs’ ability to care for them safely, and trusting them to maintain their safety. A lack of energy due to their illness was one reason for not participating in nursing care, as it was difficult for patients even to accomplish minor tasks. Some participants stated they were confused. If they were presented with different care and treatment options to choose from, they found it challenging to make the right decision.

*I’m very weak and have no strength to do anything.*

Some participants said that HCPs simply took over their care. They felt no need to participate because they felt the care they were receiving was appropriate. Some participants considered medical and nursing care was none of their business, and they did not want to interfere with something for which they were not educated. They trusted the nurses and physicians to maintain their safety and found it better to do as they were told as a patient.

*I didn’t need anything else just then because I sensed that he cared, he had taken over/ taken care of me.*

### Significance of participation

This theme included the positive and negative results of participation.

#### Feeling safe

Positive results were described by a few participants, including feeling safe and recognised when actively participating in their own care. Some stated that when they could express their feelings and thoughts freely, they felt understood by the staff, which also made them feel safer and an active member of the healthcare team. When nurses gave them opportunities to participate, participants described this as the nurses’ genuine interest in them, ultimately helping them feeling safe. Coming back to the same ward, where they were known as a patient, also gave participants a sense of security and a maintenance of safety.

*You feel safe when you participate and know what will happen to you.*

#### Feeling ignored

Negative results arose when participants felt ignored or if they thought staff were uninterested in how they felt. Some participants described how the staff failed to listen properly or did not allow time for the patients to express themselves.

*As it happens, you are feeling miserable, but they (HCPs) don’t have the time to talk to you about it.*

Another example of feeling ignored was when the participants were not given the chance to discuss with whom they share a room. Some wards placed men and women in the same rooms, which made some participants uncomfortable. Further, they felt that staff were not capable to individualising care in certain situations, such as having to go to sleep at the same time as other patients, not being able to read a book or not being able to have a light on at night. They felt ignored when staff did not recognise their needs for privacy. For instance, sharing a room with an unknown man gave one female participant a feeling of less privacy.

*This mixing of men and women in the same room, I do not like it.*

## Discussion

The importance of patient participation becomes clear in our results not only to practical participation in care but also to decision-making and communication about care. This study identified that Swedish patients wanted to play an active role in their care. Participating in care and safety activities was an opportunity for patients to be part of the decision-making process and to share information about their care and treatment. Participants believed by participating that they had the best understanding of what was happening to them and they wanted to ask questions and prevent mistakes. Family was also important for supporting and informing participants about their care and condition. This finding is in line with other research that found patients want an active role and to be seen as a partner in their care [22–26].

Power imbalances between HCPs and patients were a barrier to patient participation. To overcome these imbalances, nurses should establish a relationship with their patients to support their patients’ preferences in healthcare [27]. Meaningful relationships between HCPs and patients, honouring the whole person and respecting individual values, preferences and desires are essential. Patients also favoured hospitals where they could take an active role in their own care [10]. The legitimate role patients have in their own care is now recognised in various policy documents [28], but implementation is challenging.

Our results illustrate that participants desired participation and an improved understanding of their healthcare, which in turn increased participation. Two of the participating units in our study had person-centred care as their policy and this might have some influence on what participants said but it was not explicit. Participation is an essential component in person-centred care. According to Ekman et al. [29], actively engaging patients does not mean patients have the full responsibility for all decisions about their care. As partners, patients and HCPs should discuss and make decisions and develop care plans etc. together. If a patient’s condition makes them unable to contribute to the care planning, the HCPs should strive to include the patient’s relatives in this process [30, 31].

Our results showed that participants were not satisfied with passive roles, instead they were, for the most part, aware of their own condition and wanted to take an active role. Person-centred care is a concept where a patient is viewed as an expert about themselves as a person, with their own will and capabilities. This type of care sees the patient as a partner on the healthcare team. If a HCP adopts this person-centred manner, they need to relinquish some of their control, sharing the power with their patients in order to promote more active partnerships that patients want according to our results [29].

Participants in our study believed their ability to participate depended on their health status at the point when participation was required. Sometimes they were not able to be active in their care and safety activities because of ill health. They were then less motivated to participate and more likely to be passive, thus being passive in their participation. This passivity is a barrier to participation in safety activities. Vaismoradi et al. [3] argue that the patients’ health conditions as well as their beliefs and experiences influence their decision to participate.

Similarly, our findings suggest that nurses need to be aware of changes in patients’ health condition and assess when patients are ready to participate more actively. Several studies have highlighted that nurses must make a fundamental shift in their nursing practice to engage and involve patients [4, 32]. Soleimani et al. [33] identified levels of participation; adhering, involving, sharing and true participation. These levels have overlap and uniqueness to each other, depending on patient involvement. Adhering was the lowest level of participation and true participation was the highest level. In our study, adhering was when participants being passive and trusted nurses to care for them. Involving, in our study, occurred when participants possessed knowledge of their illness. Sharing occurred when participants did participate in specific care. Based on the study design, true participation was difficult to recognise in our study.

The results from our study showed that patients possessing knowledge about their illnesses was important for participants to participate in safety activities. Participants also wanted to be informed about their condition, laboratory tests, changes and care plans and to be continuously updated. Participants described how they generally understood their condition, but they required information on what was occurring to them during their hospitalisation. Health literacy provides individuals with an understanding about their health and enables them to access, understand, appraise and apply information to make better informed decisions about their health and healthcare [34]. A literature review revealed that low individual health literacy could lead to higher health costs for society [35]. However, patients also recognised their responsibility to share information about their health in the care plan and to make sure that the plan was appropriate and safe care was delivered [36].

Participants believed they needed to “speak up” to protect their needs and safety, which may have been supressed by a power imbalance. When nurses did not involve patients in discussions about their care, there was a power imbalance, and patients were unable to change the situation. Nurses not paying attention to patients’ wishes to take some control became a barrier to participation. Henderson (2003) [37] discovered that power imbalances arose when nurses were unwilling to share the decision-making responsibility with the patients. There are several safety campaigns encouraging patients to speak up for their own safety, yet the challenges this poses to both patients and HCPs is less recognised [38]. The shift in healthcare from hierarchal-orientated care relationship to more partnership care has emerged due to its positive impact on safety, quality of care and patient satisfaction. It also seems to be an effective model for care delivery [39]. However, much remains unknown about patient engagement in patient safety activities and what activities are the best and most effective for patients to be involved in [8].

Participants in our study reported they felt safe while being cared for in the hospital. Feeling safe meant being recognised and acknowledged as a person with their present condition by HCPs. Feeling safe promoted patient empowerment, which was linked to a feeling of being valued, a motivator for participation [40]. The concept of feeling safe has been described as “an emotional state where the perception of care contributed to a sense of security and freedom from harm”. Trust, care presence and knowledge are attributes of feeling safe [41], which lead to more patient participation in their own care and safety activities in our study.

A negative result of non-participation was that participants felt ignored. This appeared when HCPs failed to listen and/or patients were not given enough time to speak. Patient preferences and experiences are increasingly acknowledged as critical underpinnings for healthcare decision-making [1]. However, patients not only reap the benefits of health provider decisions but also bear the burden of poor decisions. Thus, patient preferences for participation are both a right and a valuable strategy for patient safety improvement.

Based on our findings, there are a number of suggestions for clinical practice in patient safety activities. Firstly, nurses but also other HCP should invite patients to participate actively in the decision-making process, ensuring they have a full understanding of the issues to be included in the process. Secondly, nurses are ideally placed to help educate patients and to be sensitive to their patients’ conditions. They should then provide more health literacy to each patient to help them make better informed decisions about their own healthcare. Thirdly, to promote better patient disclosure of medical information, HCPs should work on developing their relationships with patients by inviting patients into the care discussions. This way of thinking about patient participation in patient safety activities can also be transferred to other clinical areas, such as surgical care units and critical care but this need further research.

### Limitations

This study has several limitations. Firstly, half of the data were collected from one site that had adopted a person-centred care approach, so caution must be taken to extrapolate our results into other clinical settings. However, the findings provide important insight in patients’ thoughts about participation and safety. Secondly, patients were interviewed in the ward, which may have influenced their willingness to speak freely. Nonetheless, interviewing patients while they are at the hospital potentially increases the accuracy of their recall for their treatment, giving a more accurate representation of their thoughts on their healthcare.

## Conclusion

This study demonstrated that patients wanted to be active participants in their own care and patient safety activities. However, a number of barriers prevented this from occurring. Power imbalances, patient lack of acuity and uncertainty all hampered participation. Finding ways to consider patients’ individual needs, preferences for participation and accommodating their wishes is challenging to nurses. Giving patients a voice empowered them to be a part of the decision-making process and encouraged them to share information which in turn enabled participation. Finally, the result of participation was a feeling of safety for the patients. Patients relied on HCP to be safe in hospitals, yet they did not always know how to actively contribute to their own safety. Nevertheless, establishing good relationships between patients and HCPs appears to be the first step towards patients feeling safe and actively participating in their care.

## Abbreviation

HCPs: Healthcare professionals
